# Chronic Cadmium Exposure *in Vitro* Causes Acquisition of Multiple Tumor Cell Characteristics in Human Pancreatic Epithelial Cells

**DOI:** 10.1289/ehp.1205082

**Published:** 2012-05-24

**Authors:** Wei Qu, Erik J. Tokar, Andrew J. Kim, Matthew W. Bell, Michael P. Waalkes

**Affiliations:** Inorganic Toxicology Group, National Toxicology Program Laboratory, Division of the National Toxicology Program, National Institute of Environmental Health Sciences, National Institutes of Health, Department of Health and Human Services, Research Triangle Park, North Carolina, USA

**Keywords:** cadmium, cancer cell qualities, pancreatic cancer, pancreatic epithelial cells

## Abstract

Background: Cancer may be a stem cell (SC)–based disease involving formation of cancer SCs (CSCs) potentially arising from transformation of normal SCs. Cadmium has been linked to human pancreatic cancer.

Objective: We studied cadmium exposure of human pancreatic ductal epithelial (HPDE) cells and whether SCs may be targeted in this process.

Methods: We chronically exposed HPDE cells to low level cadmium (1 μM) for ≤ 29 weeks. Nonadherent spheroid formation was used to indicate CSC-like cell production, and we assessed tumor cell characteristics in such spheres. Assessed tumor cell characteristics including secretion of matrix metalloproteinase-9 (MMP-9), invasion, and colony formation were fortified by evaluating expression of relevant genes by real-time reverse transcription polymerase chain reaction and by Western blot.

Results: Increased MMP-9 secretion and overexpression of the pancreatic cancer marker *S100P* occurred in chronic (29 weeks of exposure) cadmium-exposed (CCE) cells. CCE cells also showed markedly higher colony formation and invasion, typical of cancer cells. Floating “spheres” of viable cells, known to contain an abundance of normal SCs or CSCs, form *in vitro* with many cell types. CCE cells produced 3-fold more spheres than control cells and were more invasive, secreted more MMP-9, and overexpressed markers for pancreatic SCs/CSCs (i.e., *CXCR4*, *OCT4*, *CD44*) and *S100P*, a marker for pancreatic cancer. CCE-derived spheres rapidly produced aggressive, highly branched, and poorly differentiated glandular-like structures in Matrigel.

Conclusions: Chronic cadmium exposure produced multiple tumor cell characteristics in HPDE cells and CCE cell–derived spheres. These data support the plausibility of cadmium as a human pancreatic carcinogen.

It is thought that cancer is a stem cell (SC)–based disease and that carcinogenesis may be a disorder of SC function (Pajonk et al. 2010; Reya et al. 2001). Emerging evidence suggests that tumor initiation, growth, progression, and metastasis are driven by cancer SCs (CSCs) (Lee et al. 2008; Tokar et al. 2011). Long-lived target tissue SCs undergo mutations or other changes that dysregulate normal SC self-renewal pathways, leading to tumor formation (Pajonk et al. 2010). CSCs have been identified in tumors of the hematopoietic system (Bonnet and Dick 1997) and in many solid tumors, including tumors of the breast (Al-Hajj et al. 2003), brain (Singh et al. 2004), colon (O’Brien et al. 2007), and pancreas (Hermann et al. 2007; Li et al. 2007). Like normal SCs, CSCs show self-renewal and are pluripotent; however, their programming and control are highly distorted (Reya et al. 2001). For reasons that are not completely clear, cultured CSCs and normal SCs often form floating spheres of viable cells enriched with SCs/CSCs (Dontu et al. 2003; Rovira et al. 2010), and this has become a method used in identification and isolation.

Cadmium and cadmium compounds are considered human and rodent carcinogens (International Agency for Research on Cancer 2012). Cadmium causes pancreatic cancer in rats (Waalkes 2003), and accumulating data suggest the human pancreas is also a target of cadmium carcinogenesis. Elevated levels of serum (Kriegel et al. 2006) or toenail (Amaral et al. 2011) cadmium correlate with human pancreatic cancer. In a meta-analysis, Schwartz and Reis (2000) found cohorts with high cadmium exposure had an increased risk of pancreatic cancer. Adams et al. (2012), using data from the Third National Health and Nutrition Examination Survey (NHANES III) cohort, detected an association between cadmium exposure and pancreatic cancer in men. However, the mechanisms of cadmium carcinogenesis at any site remain undefined, and additional models at all levels of biological complexity, including models of pancreatic cancer, are clearly needed.

Pancreatic cancer is a leading cause of cancer-related death (Jemal et al. 2008) and is characterized by early metastasis and late diagnosis (Gilbert and Ross 2009), giving it a generally poor clinical prognosis (Hermann et al. 2009). CSCs have been identified in human pancreatic cancers and play a key role in tumor progression and metastasis (Hermann et al. 2007; Li et al. 2007). Several markers are used to identify pancreatic CSCs. For instance, *CD44*, a stem cell surface marker, is highly expressed in a tumorigenic subpopulation of pancreatic cancer cells (Li et al. 2007). A subpopulation of CSCs overexpressing the cytokine receptor 4 (*CXCR4*) is essential for pancreatic tumor metastasis (Hermann et al. 2007). The gene for octamer-binding transcription factor 4 (*OCT4*) is considered a pluripotency gene required for the self-renewal and maintenance of SCs, and most CSCs express high levels of this factor (Sotomayor et al. 2009). In this regard, *OCT4* expression occurs in adult human pancreatic SCs during differentiation (Tai et al. 2005). Non-SC markers for pancreatic cancer such as S100P, which is a small calcium binding protein with diverse normal regulatory functions and also promotes pancreatic cancer growth, tumor cell survival and invasion, are overexpressed in pancreatic cancers (Arumugam et al. 2005).

Functional assays such as sphere-formation capacity *in vitro* can also be important for identifying SCs or CSCs. For instance, isolated human mammary SCs commonly generate nonadherent spheroids in culture called “mammospheres,” which are highly enriched in mammary SCs and capable of both self-renewal and differentiation (Dontu and Wicha 2005). Numerous other examples exist of SCs from other tissues forming spheres enriched in SCs *in vitro* (Reynolds and Weiss 1996; Tokar et al. 2010a). CSCs isolated from various human pancreatic cancer cells, like SCs, also produce nonadherent spheres *in vitro* (Gaviraghi et al. 2010; Gou et al. 2007). This is similar to breast and hematopoietic systems where isolated CSCs produce spheres (Al-Hajj et al. 2003; Bonnet and Dick 1997). However, little is known about the effects of chemical carcinogens on pancreatic sphere-forming capacity or qualities.

Thus, because cadmium exposure is linked with human pancreatic cancer, in the present study we first determined whether chronic cadmium exposure *in vitro* could cause human pancreatic epithelial cells to acquire tumor cell characteristics. For this we selected a well-characterized human pancreatic ductal epithelial (HPDE) cell line (Furukawa et al. 1996). After demonstrating that chronic low-level, nontoxic cadmium exposure caused acquisition of various characteristics typical of pancreatic cancer cells (e.g., increased matrix metalloproteinase secretion, colony formation, invasion), we studied the nature and quantity of spheroids produced after cadmium exposure because these have been reported to be produced by CSCs in culture.

## Materials and Methods

***Chemicals and reagents.*** We purchased cadmium chloride, chloroform, isopropanol, and formaldehyde from Sigma-Aldrich Chemical Company (St. Louis, MO). A cell proliferation assay kit obtained from Promega (Madison, WI) based on the 3-(4,5-dimethylthiazol-2-yl)-5-(3-carboxymethoxyphenyl)-2-(4-sulfophenyl)-2H-tetrazolium, inner salt (MTS) assay was used according to the manufacturer’s instructions. Antibodies were purchased from the following suppliers: anti-S100P (BD Biosciences Pharmingen, San Jose, CA); anti-OCT4 (Santa Cruz Biotechnology Inc., Santa Cruz, CA); anti-CXCR4, (Calbiochem, San Jose, CA); and anti-CD44 (Abcam Inc., Cambridge, MA).

***Cell culture and treatment***. The HPDE cell line, originally derived from normal adult human pancreas and is immortalized and nontumorigenic (Furukawa et al. 1996), was graciously supplied by M.-S. Tsao, University of Toronto. Cadmium chloride was used in all studies that involved cadmium exposure. We cultured HPDE cells in complete keratinocyte serum-free medium supplemented with 50 μg/mL bovine pituitary extract (BPE), 5 ng/mL epidermal growth factor (EGF), and 1% antibiotic/antimycotic mixture (Gibco/Invitrogen, Rockville, MD) at 37^o^C in a humidified incubator containing an atmosphere of 5% carbon dioxide/95% air. Control cells were cultured with passage once per week in unaltered medium and served as a passage-matched control, whereas 1.0 μM cadmium was added to induce acquisition of cancer cell properties. We selected the 1.0-μM level of cadmium because it was nontoxic in HPDE cells in preliminary study. Cadmium is a bioaccumulative metal and the level chosen for this *in vitro* study [1.0 μM (112 μg/L)] is well below that reported in the pancreas of adult persons living in non-cadmium–polluted areas of Japan [7.4–10.5 mg/kg wet weight; Uetani et al. (2006)]. Cell samples were frozen every 2 weeks for later use. We cultured A549 cells in Dulbecco’s modified Eagle’s medium containing 10% fetal bovine serum.

***Cell proliferation***. We assessed cell proliferation using the Promega cell proliferation kit. The lethal concentration 50% (LC_50_) values of cadmium were determined by analysis of the linear portion of multiple metabolic integrity curves.

***Assessment of acquired cancer cell characteristics***. We assessed several characteristics common to tumor cells during chronic cadmium exposure. This included zymographic analysis of matrix metalloproteinase-9 (MMP-9) secretion as described (Achanzar et al. 2002). Cellular invasiveness was examined using a modified Boyden chamber invasion assay (Tokar et al. 2010a). We used the human lung carcinoma cell line A549 as a positive control for the cellular invasion studies. We performed colony formation in soft agar as described (Tokar et al. 2010b). The formation of colonies of cells with the capacity for anchorage-independent growth in agar is accepted as a reflection of cancer phenotype.

***Floating sphere formation***. Based on increased secretion of MMP-9, invasiveness, colony formation, and other genetic factors, the chronic cadmium-treated cells had acquired numerous qualities typical of pancreatic cancer cells after 29 weeks of exposure. At this point, we designated them as chronic cadmium-exposed (CCE) cells to reflect these cadmium-induced changes. In order to obtain cells that form nonadherent spheroids, we collected floating cell suspensions from cadmium or control culture medium by gentle centrifugation (1,000 rpm, 5 min). We plated equal numbers of control and treated cells (1,000) in ultralow attachment plates (Corning Inc., Corning, NY) for 10 days with feeding every 72 hr. Under these conditions, floating spherical colonies are generated in culture, which we termed pancreaspheres. To avoid breaking up the spheres, fresh medium was gently added to each flask and no medium was aspirated. Cadmium treatment continued during this period.

***Gene expression and immunoblotting***. We measured transcriptional expression by quantitative real-time reverse transcription polymerase chain reaction (RT-PCR). Total RNA was isolated from cells using TRIzol (Invitrogen, Carlsbad, CA) and purified with Qiagen RNeasy mini kit columns (Qiagen, Valencia, CA). Total RNA from each sample was reverse transcribed with MuLV reverse transcriptase and Oligo d(T) primers (Applied Biosystems, Foster City, CA). We used SYBR Green PCR Kits (Applied Biosystems) for analysis. Primers were designed using Applied Biosystems Primer Express software [for a list of sequences used in real-time RT-PCR, see Supplemental Material, Table S1 (http://dx.doi.org/10.1289/ehp.1205082)]. Relative differences in gene expression between groups were expressed using cycle time (Ct) values first normalized with that of β-actin in the same sample and expressed as arbitrary units. Real-time fluorescence detection was carried out using a MyiQ™ singleColor Real-Time PCR Detection System (Bio-Rad, Hercules, CA).

We determined protein expression by Western blot analysis. Cells were lysed by adding 1× SDS sample buffer with 1% Protease Inhibitor Cocktail and 1% Phosphatase Inhibitor Cocktail 1 (Sigma-Aldrich). Adherent cells were scraped off the plate and transferred to a microcentrifuge tube on ice. For sphere cells, all culture medium was centrifuged at 2,000 rpm for 10 min, and lysis buffer was added to the cell pellet. The sample was sonicated for 10–15 sec, centrifuged at 14,000 rpm (10 min) and an aliquot of supernatant (containing 30 µg protein) was taken. For Western blot analyses, membranes were incubated with the S100P primary antibody (1:2,000 dilution), washed and incubated with horseradish peroxidase–conjugated anti-mouse IgG secondary antibody (1:2,000 dilution; New England Biolabs, Beverly, MA). Immunoblots were visualized by the LumiGlo detection method (New England Biolabs). Membranes were then stripped with Restore Western Blot Stripping Buffer (Pierce Biotechnology, Rockford, IL) and incubated with anti–β-actin antibody (mouse monoclonal, 1:5,000 dilution; Sigma-Aldrich) followed by incubation with horseradish peroxidase–conjugated anti-mouse IgG secondary antibody (1:2,000 dilution; New England Biolabs). Relative densities of the bands were digitally quantified using ImageJ software (http://download-imagej.com/).

For immunostaining for co-localization of proteins, we cultured cells on chambered coverglass (Nalge Nunc International, Rochester, NY), fixed them with methanol and acetone (1:1) for 2 min, washed them with PBS, and preincubated them with blocking buffer containing 5% milk, 0.1% gelatin, 7.5% sucrose, and avidin-biotin blocking reagents (Vector Laboratories Inc., Burlingame, CA) in PBS. Fixed cells were incubated with primary antibodies (1:500) against CXCR4, OCT4, CD44, and S100P for 1 hr, washed three times, stained with Alexa Fluro 568– and 488–conjugated secondary antibodies (1:1,000; Molecular Probes, Eugene, OR) for 30 min, and washed three times with PBS. The nuclei of the cells were stained with 4´-6-diamidino-2-phenylindole (DAPI; Molecular Probes) for 5 min each. We examined slides using DP72-BSW microscope digital camera software (Olympus America Inc., Center Valley, PA).

***Formation of branched ductal-like structures in Matrigel***. As described (Webber et al. 1997), we added 250 µL of Matrigel to the wells of 48-well plates and incubated them at 37^o^C for 1 hr. Pancreaspheres were plated on the gel, then 250 µL of Matrigel was gently layered above, followed by 300 µL of complete keratinocyte serum-free medium. The plates were then incubated at 37^o^C for 20 days with fresh medium every 5–7 days. Growth of glandular-like structures was periodically assessed and photographed. After 20 days, gels were washed gently with PBS, fixed in 4% formaldehyde for 15 min, rinsed twice with PBS, and either stained as whole mounts with hematoxylin and eosin to assess gross morphology or embedded in paraffin, fixed, sectioned, and stained for light microscopy.

***Statistical analysis***. We expressed data as mean ± SE. We used Student’s *t*-test or analysis of variance with subsequent Dunnett’s test as appropriate. Values are derived from three or more replicates. We considered differences significant at a level of *p* < 0.05.

## Results

**Figure 1:**
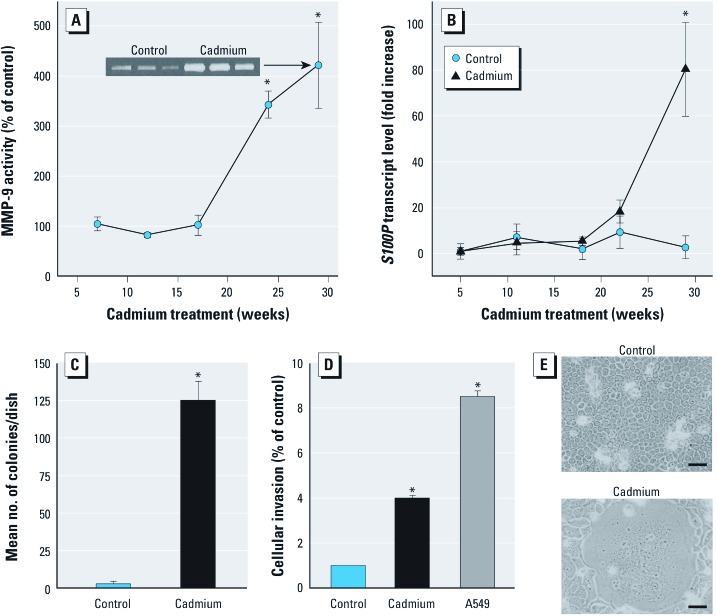
Chronic cadmium exposure and acquired cancer cell characteristics in HPDE cells. (*A*) Secreted MMP-9 activity from control and cadmium-treated HPDE cells; inset, representative zymogram (29 weeks). (*B*) *S100P* transcript levels. (*C*) Colony formation in soft agar; mean number of colonies per dish was 3 (control) and 126 (29 weeks of cadmium exposure). (*D*) Invasion; the A549 human lung adenocarcinoma cell line was used as a positive control. (*E*) Cadmium-induced morphological changes (see text for description); control cells (top) and cadmium-treated cells (bottom); magnification, ×200; bars = 50 µm. Data represent mean ± SE (*n* = 4). *p < 0.05 compared with control.

***Chronic cadmium treatment induces multiple cancer cell characterstics in HDPE cells***. Previous work showed that increased secretion of MMP-9 is associated with cadmium-induced malignant transformation of human epithelial cells (Achanzar et al. 2001). Therefore, we assayed secreted MMP-9 activity every 4–5 weeks in HPDE cells exposed to nontoxic cadmium levels. MMP-9 secretion was markedly increased by 24 weeks, peaking at 29 weeks of cadmium exposure (Figure 1A). A member of the family of small calcium binding proteins, S100P can mediate tumor growth and metastasis and is highly expressed in pancreatic cancers but low levels are found in the normal pancreas or with chronic pancreatitis (Arumugam et al. 2004). To help further define whether chronic cadmium exposure induced characteristics typical of pancreatic cancer cells, S100P levels were determined during cadmium exposure. Expression of *S100P* increased markedly at 29 weeks of cadmium exposure (Figure 1B). Chronic cadmium exposure increased colony formation (Figure 1C) and cellular invasion (Figure 1D). The human lung adenocarcinoma cell line A549 was used as a positive control for invasion (Figure 1D). Chronic cadmium exposure induced morphological changes, including loss of contact inhibition, atypical foci of cell mounding, and formation of giant multinuclear cells (Figure 1E), which are common with cancer cells. Based on a variety of quantitative and qualitative factors, including increased MMP-9 secretion, increased invasiveness, overexpression of *S100P*, increased colony formation, and morphological changes, exposing HPDE cells to cadmium for 29 weeks caused acquisition of multiple tumor cell characteristics and these are henceforth designated CCE (chronic cadmium exposed) cells to reflect this distinction from control cells.

**Figure 2:**
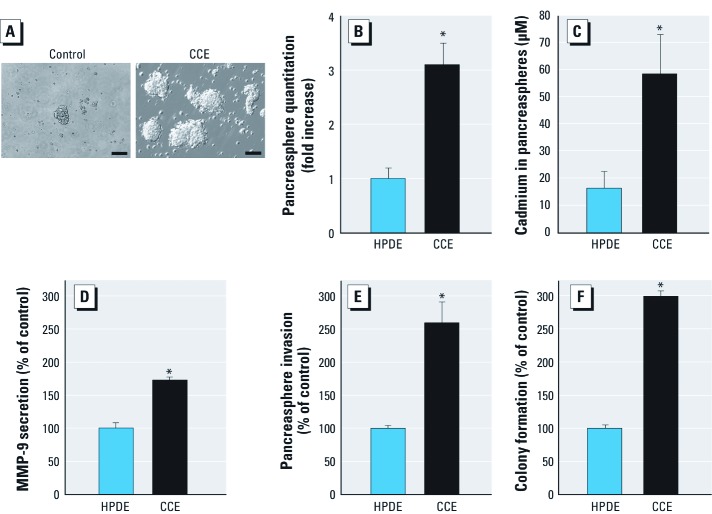
CCE- or control cell–derived pancreasphere formation and their characteristics. (*A*) Free-floating, viable spheres from control (left) or CCE cells (right); magnification, ×100; bars = 50 µm. (*B*) Pancreasphere quantitation derived from CCE (29 weeks) or control cells. (*C*) Cadmium concentration lethal to 50% of cells (LC_50_) derived from survival curves. (*D*) Secreted MMP-9 activity from pancreaspheres derived from CCE or control cells. (*E*) Invasion by pancreasphere cells derived from CCE or control cells. (*F*) Colony formation in soft agar assay with pancreaspheres derived from CCE or control cells. Data represent mean ± SE (*n* = 3). *p < 0.05 compared with control.

***Cadmium exposure and pancreasphere formation***. To determine whether chronic cadmium exposure resulting in acquisition of multiple cancer cell characteristics stimulates pancreasphere formation, we studied the formation of floating spheres from the same number of CCE and control cells, first determining size and number (Figure 2A,B). Prior work indicates that these pancreaspheres are likely free floating clusters of viable cells enriched with SCs (control) or CSCs (tumors). CCE cell–derived pancreaspheres typically grew larger than control spheres over the same time period (Figure 2A). Quantitatively, CCE cells produced 3-fold more pancreaspheres than control cells (Figure 2B). Further, CCE cell–derived pancreaspheres showed resistance to acute cadmium cytolethality compared to control cell–derived spheres, as reflected in a higher LC_50_ (Figure 2C). MMP-9 secretion was increased in CCE cell–derived pancreaspheres compared to control cell–derived spheres (Figure 2D). Invasion of CCE cell–derived pancreaspheres was higher than control cell–derived spheres (Figure 2E). Colony formation increased in CCE cell–derived sphere cells compared to control cells (Figure 2F). These last three qualities (increased secretion of MMP-9, invasiveness, and colony formation) are consistent with cancer cells. Cancer cell qualities would be expected with a CSC-enriched pancreasphere, but are not absolute proof these CCE cell–derived spheres contain CSCs.

**Figure 3:**
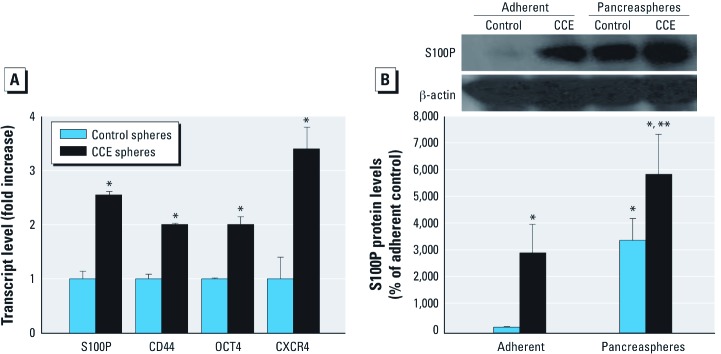
Expression of genes associated with CSCs/SCs and/or pancreatic cancer in CCE- or control cell–derived pancreaspheres. (*A*) Transcript level of CSC/SC genes in pancreaspheres derived from CCE or control cells. (*B*) Expression of S100P protein in both adherent cells and pancreaspheres derived from CCE or control cells by Western blot analysis; blot represents a typical result of three independent experiments (*B*, top). After development, the membranes were stripped and reprobed with β-actin as the control for equal protein loading (*B*, middle). Then, immunoblots were analyzed by scanning densitometry and values were standardized to adherent control (*B*, bottom). Data represent mean ± SE (*n* = 3). *p < 0.05 compared with control adherent cells. **p < 0.05 compared with control pancreaspheres.

***CSC and pancreatic cancer markers in pancreaspheres***. Markers for CSCs/SCs or pancreatic cancer, including *S100P*, *OCT4*, and *CXCR4*, were assessed in pancreaspheres. The transcriptional expression of these various markers was increased in CCE cell–derived spheres compared to control cell–derived spheres (Figure 3A). This was confirmed at the protein level for S100P (Figure 3B) when cadmium markedly increased S100P protein in adherent CCE cells and increased it even more in CCE cell–derived pancreaspheres.

**Figure 4:**
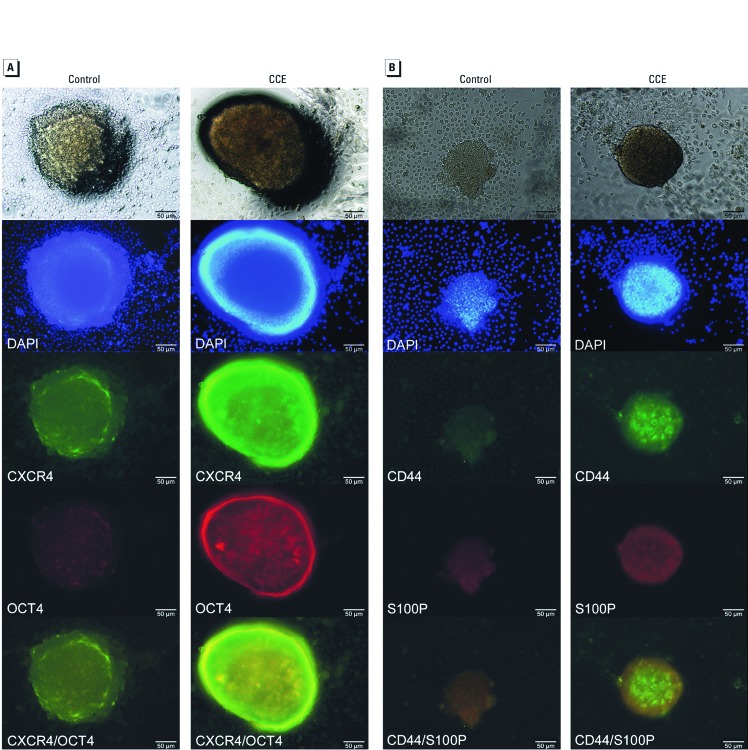
Expression of genes associated with CSCs/SCs and/or pancreatic cancer in CCE- or control cell–derived pancreaspheres assessed by immunohistochemical imaging. (*A*) Fluorescence microscopic imaging of CXCR4 and OCT4 protein in pancreaspheres. (*B*) Expression of CD44 and S100P protein in pancreaspheres. The top row of images show unstained pancreaspheres. The second row of images show DAPI-stained nuclei, indicating viable cells. The third and fourth row of images show, respectively, staining with Alexa Fluor 488– or 568–conjugated secondary antibodies. The bottom row shows merged third- and fourth-row images to illustrate co-localization of proteins. The primary antibodies used are indicated under each image. All images are the same magnification (×200); bars = 50 µm. Data are representative of four separate experiments.

***Immunohistochemical evidence of enhanced expression of CSC markers in pancreaspheres***. Pancreaspheres were immunostained with CXCR4 and OCT4 antibodies, the nuclei were counterstained with DAPI, and the images were merged (Figure 4A). CCE cell–derived pancreaspheres had much higher expression of both CXCR4 and OCT4 proteins compared to control spheres as well as greater co-localization within the spheres. Similarly, CCE cell–derived pancreaspheres also showed higher expression and co-localization of CD44 and S100P proteins compared to control spheres (Figure 4B).

**Figure 5:**
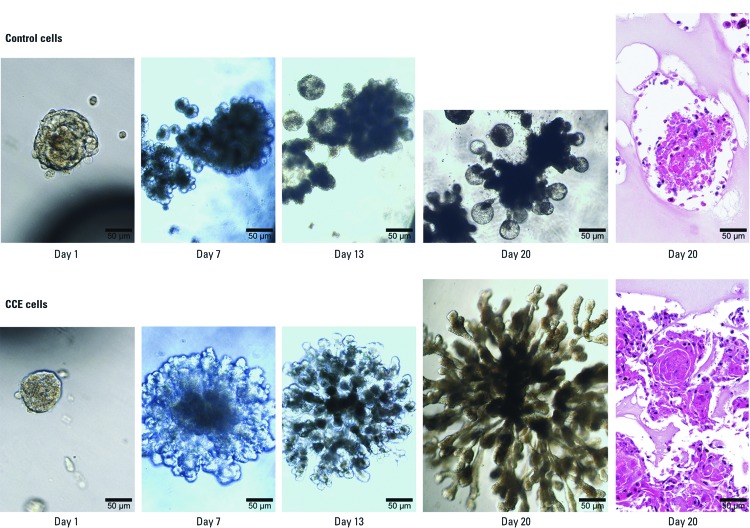
Ductal/glandular-like structures produced by CCE- or control cell–derived pancreaspheres. Single pancreaspheres from control cells (top) and CCE cells (bottom) were selected and placed on Matrigel. The first four panels (days 1, 7, 13, 20) show the progressive morphologic changes towards branched glandular-like structures that were observed *in situ* using light microscopy. Additional pancreaspheres were grown in Matrigel for 20 days, then fixed, sectioned, stained with H&E (far right; 20 days), and examined using light microscopy. Note clusters of poorly differentiated cells in these CCE sphere–derived structures. All images are of the same magnification (×200); bars = 50 µm. Data are representative of four separate experiments.

***Growth of pancreaspheres in Matrigel***. Formation of ductal-like structures in Matrigel is typical for SCs from various tissues. Morphologically progressive changes typical for such culture of single CCE- and control cell–derived pancreaspheres are shown in Figure 5. CCE cell–derived pancreaspheres grew more rapidly, producing more highly branched glandular-like structures than control spheres. CCE-cell sphere–derived glandular structures appeared to be much more aggressive, with many more protrusions. In stained cross-section, these glandular-like structures from CCE cell–derived pancreaspheres appeared to be much more malignant, with multiple poorly differentiated cells, and cells of various sizes and shapes, for example. This is consistent with chronic cadmium exposure causing acquisition of multiple cancer cell characteristics in CCE cells and CCE cell–derived pancreaspheres. Pancreaspheres are known to contain SCs or CSCs, depending on their cell of origin.

## Discussion

In support of accumulating human data associating cadmium exposure with pancreatic cancer (Adams et al. 2012; Amaral et al. 2011; Kriegel et al. 2006; Schwartz and Reis 2000), the present study shows chronic *in vitro* exposure to a nontoxic, low level of cadmium causes acquisition of multiple tumor cell characteristics in normal human pancreatic epithelial cells. In this regard, a variety of characteristics typical of tumor cells were found in CCE cells. MMPs are often overexpressed by neoplastic pancreatic epithelium (Jaffee et al. 2002), and MMP-9, an enzyme secreted by cancer cells to digest extracellular matrix and aid invasion and metastasis, was markedly increased in the CCE cells. *S100P* is overexpressed in most pancreatic cancers and plays a major role in tumor aggressiveness (Arumugam et al. 2005) and was highly increased in CCE cells. CCE cells were also highly invasive and showed higher colony formation and loss of contact inhibition, all characteristics typical of tumor cells. These results help fortify the human data associating cadmium and pancreatic cancer by indicating that cadmium exposure can influence human pancreatic epithelial cells *in vitro* to acquire multiple characteristics of cancer cells.

Pancreatic cancer is insidious; its incidence and mortality rates are almost identical because it is generally first diagnosed after metastasis (Tonini et al. 2007). Therefore, it is important to define the very early stages of pancreatic cancer in order to detect and prevent new cancers. In this regard, the CSC hypothesis is an attractive model to explain tumor initiation, growth, progression, and metastasis. In this theory, cancer development and propagation is mediated by a small subset of highly tumorigenic CSCs within a tumor (Pajonk et al. 2010). Tumor cells are heterogeneous, but presumably only CSCs can form or reform tumors (Pajonk et al. 2010). The existence of CSCs has been demonstrated in human pancreatic cancers, where they appear to play a role in tumor promotion, progression, and metastasis (Hermann et al. 2007; Li et al. 2007). Generally speaking, CSCs often have been identified and isolated by their sphere-forming capacity (Dontu and Wicha 2005). In the present study, chronic treatment of pancreatic epithelial cells with cadmium markedly increased pancreasphere formation, and these spheres had multiple cancer cell characteristics typical of CSCs. CCE cell–derived pancreaspheres were highly resistant to cadmium, showed increased secretion of MMP-9, and had increased invasiveness and colony formation compared with control-derived spheres. Spheres derived from CCE cells generated more aggressive and complex glandular structures in Matrigel than those from control cells. This aggressive nature was confirmed by molecular analysis showing that CCE cell–derived spheres overexpressed common CSC or pancreatic cancer markers. Overexpression of the cell surface antigen *CD44* is considered to be a pancreatic CSC marker that acts as an adhesion molecule with multiple signaling functions (Lee et al. 2008). *OCT4* is a SC marker critical for self-renewal and is overexpressed in human pancreatic cancer cells (Iki and Pour 2006). Adult SCs maintain expression of *OCT4*, and because CSCs express this gene, this suggests tumor cells are derived from normal SCs (Tai et al. 2005). *CXCR4* is a key regulator of tumor invasiveness, progression, and metastasis (Burger and Kipps 2006), and CXCR4-positive CSCs appear to represent an invasive population with migratory activity (Hermann et al. 2007). Thus, chronic cadmium exposure *in vitro* of human pancreatic cells causes increased formation of more aggressive pancreaspheres. These spheres derived from CCE cells showed multiple cancer cell characteristics. Others (Chen et al. 2012; Ghods et al. 2007) have shown spheres derived from cancer cell lines are enriched in CSCs, although the nature of the CCE sphere cells requires further work to define.

*S100P* is commonly overexpressed in human pancreatic cancer (Arumugam et al. 2005). Silencing of *S100P* decreases pancreatic cancer cell growth, motility, invasion, and survival *in vitro*, whereas *S100P* overexpression increases pancreatic cancer cell aggressiveness and is associated with increased pancreatic tumor growth and metastasis *in vivo* (Arumugam et al. 2005; Pajonk et al. 2010). In the present study, *S100P* expression was markedly increased in CCE cells. In addition, CCE cell–derived spheroids also overexpressed *S100P*. Thus, *S100P* expression likely contributes to the aggressive nature of these CCE cells and CCE cell–derived speroids.

## Conclusions

Chronic cadmium exposure *in vitro* caused the acquisition of multiple cancer cell characteristics in human pancreatic epithelial cells. In addition, CCE cells produced nonadherent pancreaspheres with an aggressive nature that also showed multiple cancer cell characteristics. These data support the plausibility of cadmium as a human pancreatic carcinogen.

## Supplemental Material

(70 KB) PDF
