# Supplementary material for: Chronic Cadmium Exposure in Vitro Causes Acquisition of Multiple Tumor Cell Characteristics in Human Pancreatic Epithelial Cells
Source: Environ Health Perspect. 2012 May 24;120(9):1265–71. doi: 10.1289/ehp.1205082 (PMC3440134; doi:10.1289/ehp.1205082)
Supplement: (70 KB) PDF [file ehp.1205082.s001.pdf]

Supplemental Material

Chronic Cadmium Exposure In Vitro Causes Acquisition of Multiple Tumor Cell Characteristics in Human Pancreatic Epithelial Cells

Wei Qu, Erik J. Tokar, Andrew J. Kim, Matthew W. Bell, and Michael P. Waalkes

Supplemental Material, Table S1: Primer sequences used for real-time RT-PCR.

Gene	Forward primer (5'→ 3')	Reverse primer (5'→ 3')
CD44	ACCCTCCCCTCATTCACCAT	CGATATCCCTCATGCCATCTG
OCT4	CCCCATTTCACCACACTCTACTC	CCAGAGCAGTGACAGGAACAGA
CXCR4	ACTGAGAAGCATGACGGACAAG	GAAGGGAAGCGTGATGACAAAG
S100P	TGCAGAGTGGAAAAGACAAGGA	CCTGGGCATCTCCATTGG
β-actin	CTGGAACGGTGAAGGTGACA	ATGGCAAGGGACTTCCTGTAAC
